# Supplementary material for: Regulation of Host Immune Response against Enterobacter cloacae Proteins via Computational mRNA Vaccine Design through Transcriptional Modification
Source: Microorganisms. 2022 Aug 10;10(8):1621. doi: 10.3390/microorganisms10081621 (PMC9415879; doi:10.3390/microorganisms10081621)
Supplement: Supplementary file 1 [file microorganisms-10-01621-s001.zip › microorganisms-1840761-supplementary.pdf]

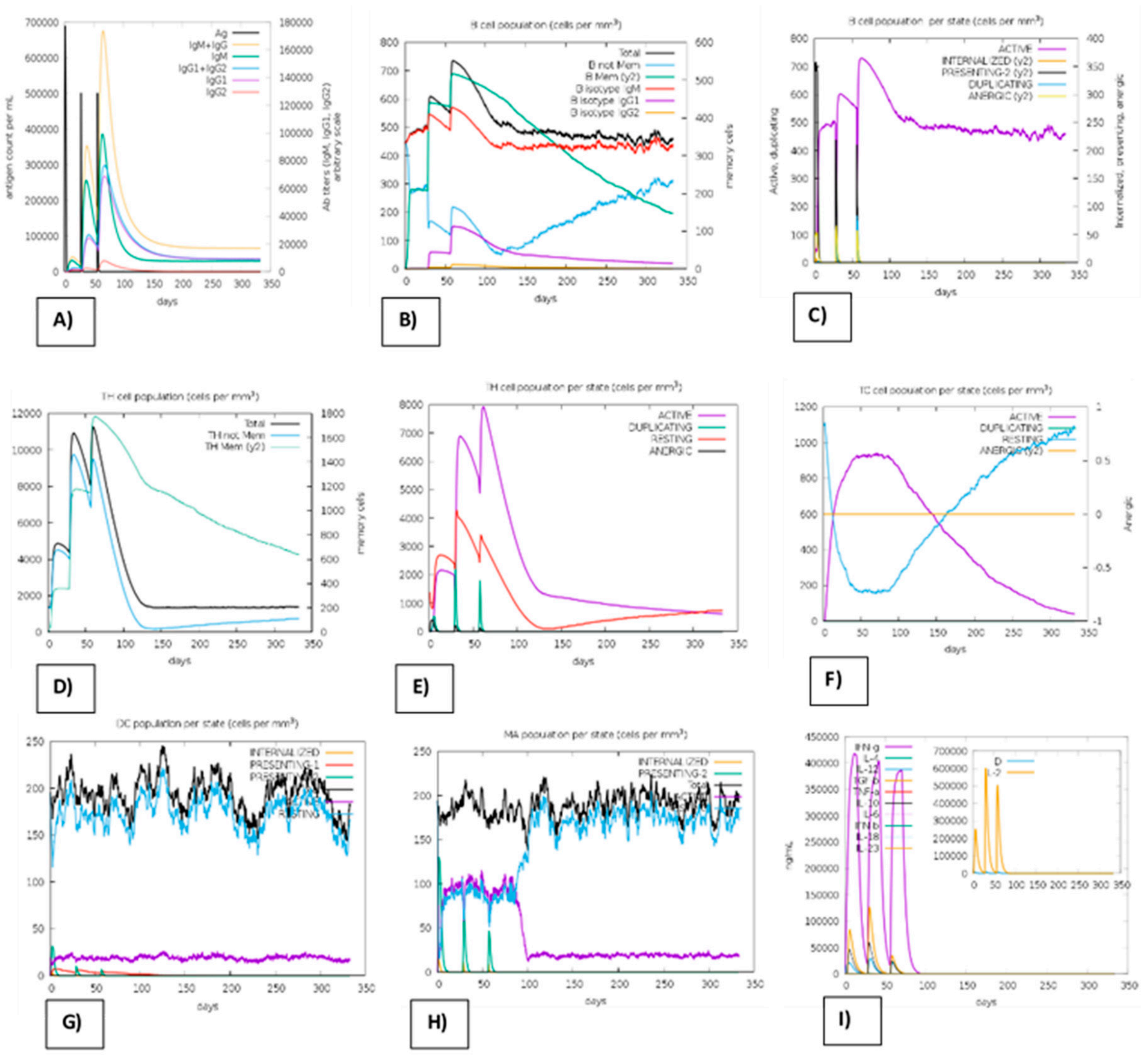

Supplementary Figure S1. Immune simulation response against Vaccine construct from C-ImmSim server. **(A)** The immunoglobulin production after antigen injection. **(B)** The B cell population after three injections. **(C)** The B Cell Population per state **(D)** The Helper T Cell Population **(E)** The Helper T Cell Population per state **(F)** The Cytotoxic T Cell Population per state **(G)** Macrophage Population per state **(H)** Dendritic Cell Population per state **(I)** Cytokines and Interleukins Production with Simpson Index of the immune response.

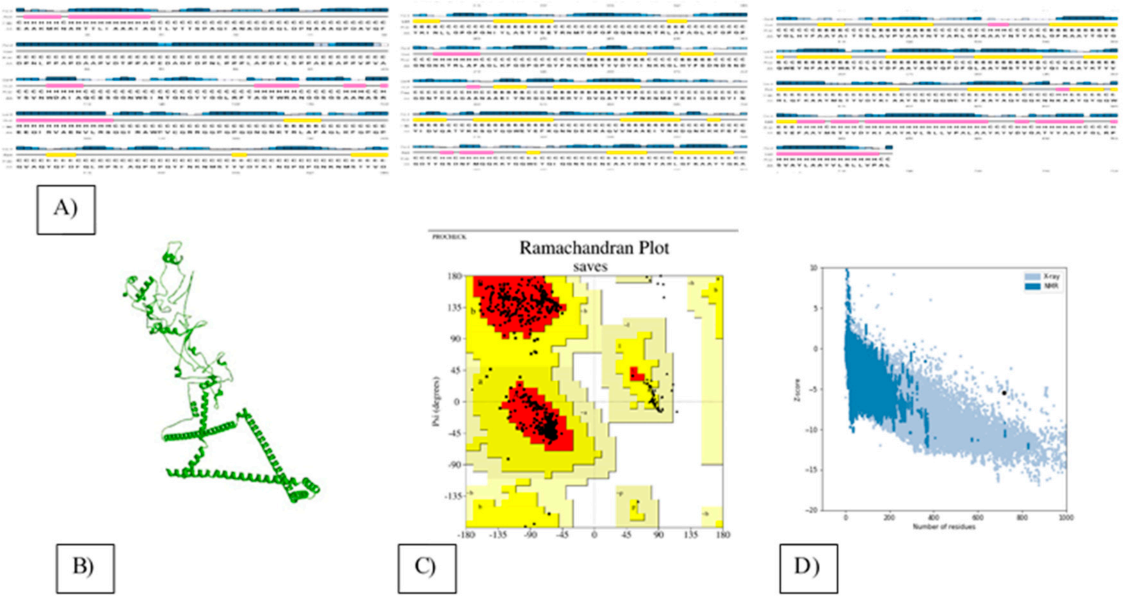

Supplementary Figure S2. Structure prediction and validation of Vaccine Construct: **(A)** PSIPRED server results of secondary structure of vaccine **(B)** The Robetta server used to predict the Tertiay structure of vaccine **(C)** The PROCHECK server used to analyze the Ramachandran Plot **(D)** Z-score analysed by the Pro-SA webserver.

I

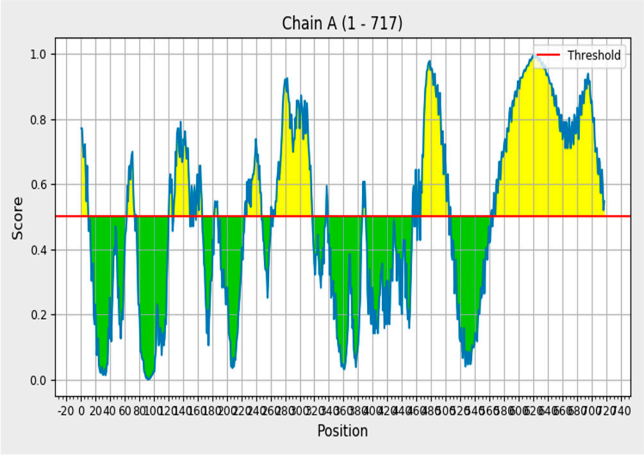

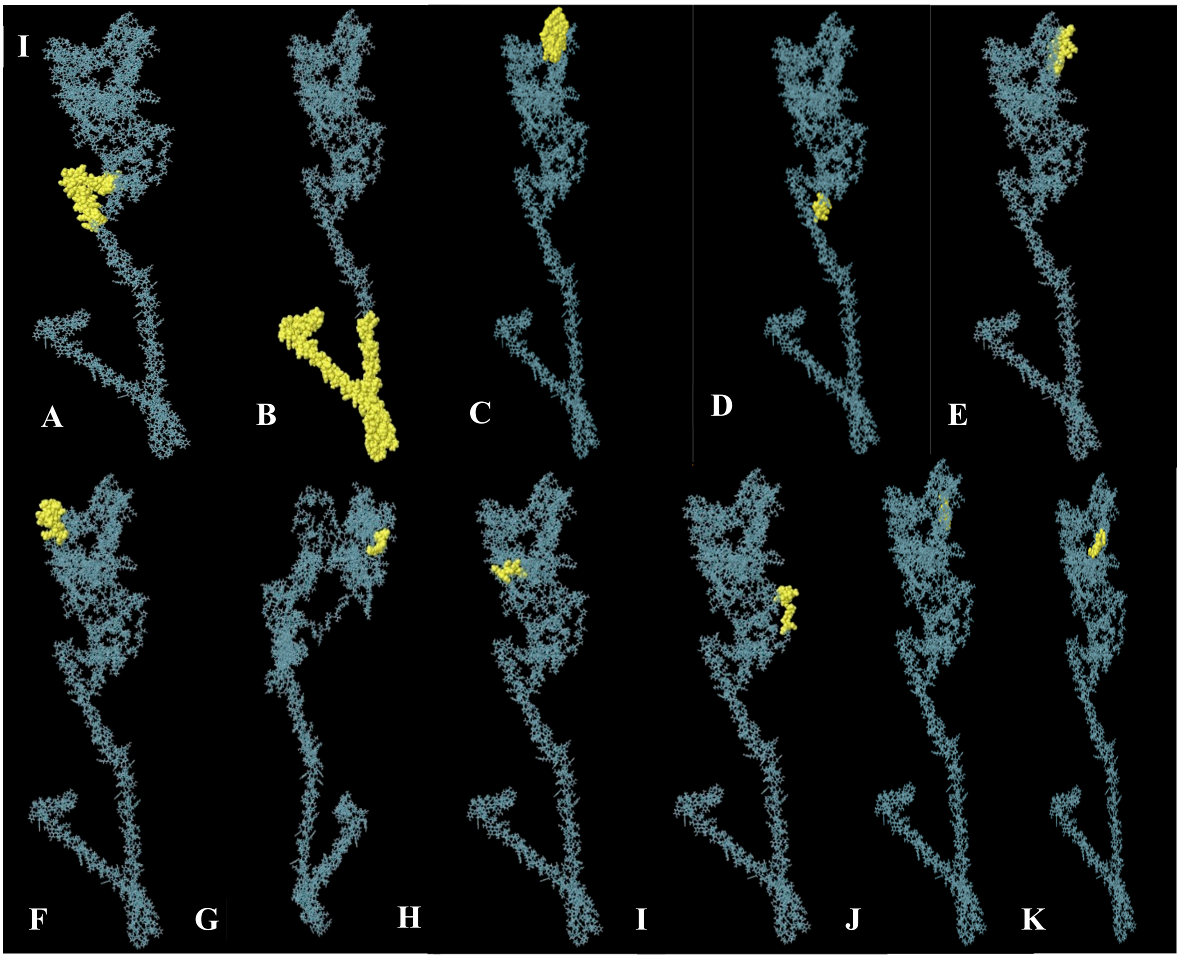

Supplementary Figure S3. The ElliPro server of IEBD database for the prediction of eleven conformational B-cell epitopes: (I) Position of Conformational B-cell epitopes 2D illustration. (II) 3D models of B-cells epitopes where yellow spheres present the conformational B-cell epitopes. **(A)**35 residues with a score of 0.819 **(B)** 155 residues with a score of 0.819 **(C)** 42 residues with a score of 0.802 **(D)** 10 residues with a score of 0.675 **(E)** 13 residues with a score of 0.634 **(F)** 51 residues with a score of 0.615 **(G)** 9 residues with a score of 0.612 **(H)**11 residues with a score of 0.579 **(I)** 20 residues with a score of 0.568 **(J)** 6 residues with a score of 0.532 **(K)** 6 residues with a score of 0.525.
